# Supplementary material for: Discovery and application of insertion-deletion (INDEL) polymorphisms for QTL mapping of early life-history traits in Atlantic salmon
Source: BMC Genomics. 2010 Mar 8;11:156. doi: 10.1186/1471-2164-11-156 (PMC2838853; doi:10.1186/1471-2164-11-156)
Supplement: Additional file 2 — Information on developed 76 locus single-run INDEL panel in Atlantic salmon. Information on fluorescence labeling, primer concentrations, PCR pooling and links to alignments, INDEL motifs and GENESCAN (Burge and Karlin 1997) predictions of genes/exons are available in html format. [file 1471-2164-11-156-S2.ZIP › Additionalfile2/snpsummary228.html]

```
Cluster 18 Contig 4

prev  Summary    Contig List  next
```

Size of Consensus sequence = 1454

Number of sequences = 139

Minimum redundancy = 6

Key

A gi|84208142|gb|DW179891.1|DW179891 SGP303495 Atlantic salmon Gills II cDNA library Salmo salar cDNA clone SS0076E01 5', mRNA sequence  
B gi|84207901|gb|DW179761.1|DW179761 SGP303355 Atlantic salmon Gills II cDNA library Salmo salar cDNA clone SS0065F06 5', mRNA sequence  
C gi|45307332|gb|CK877701.1|CK877701 SGP139034 Atlantic salmon Gills cDNA library Salmo salar cDNA clone G6-1078 5', mRNA sequence  
D gi|57122274|gb|CX353715.1|CX353715 ssalrgb523347\_rev\_0 mixed\_tissue Salmo salar cDNA, mRNA sequence  
E gi|84207943|gb|DW179789.1|DW179789 SGP303384 Atlantic salmon Gills II cDNA library Salmo salar cDNA clone SS0066C01 5', mRNA sequence  
F gi|84208394|gb|DW180020.1|DW180020 SGP303632 Atlantic salmon Gills II cDNA library Salmo salar cDNA clone SS0106C05 5', mRNA sequence  
G gi|84208168|gb|DW179904.1|DW179904 SGP303509 Atlantic salmon Gills II cDNA library Salmo salar cDNA clone SS0076H12 5', mRNA sequence  
H gi|45315024|gb|CK885393.1|CK885393 SGP165765 Atlantic salmon Intestine cDNA library Salmo salar cDNA clone T4-1404 5', mRNA sequence  
I gi|84208311|gb|DW179976.1|DW179976 SGP303586 Atlantic salmon Gills II cDNA library Salmo salar cDNA clone SS0104E09 5', mRNA sequence  
J gi|29314365|gb|CB503139.1|CB503139 ssalplnb510108\_rev gut Salmo salar cDNA, mRNA sequence  
K gi|84984039|gb|DW534389.1|DW534389 EST\_ssal\_plnb\_3025 plnb Salmo salar cDNA clone ssal\_plnb\_020\_186\_rev 5', mRNA sequence  
L gi|45307098|gb|CK877467.1|CK877467 SGP138783 Atlantic salmon Gills cDNA library Salmo salar cDNA clone G6-0460 5', mRNA sequence  
M gi|45323215|gb|CK893482.1|CK893482 SGP153569 Atlantic salmon Spleen cDNA library Salmo salar cDNA clone MI6-0122 5', mRNA sequence  
N gi|84971342|gb|DW469743.1|DW469743 SGP298622 Atlantic salmon Testis cDNA library Salmo salar cDNA clone MG4-2912 5', mRNA sequence  
O gi|29328031|gb|CB516805.1|CB516805 ssalrgb509310\_rev mixed\_tissue Salmo salar cDNA, mRNA sequence  
P gi|15846322|gb|BG936494.1|BG936494 SS1-0869 Atlantic Salmon spleen Salmo salar cDNA clone SS1-0869 similar to MHCII beta chain, mRNA sequence  
Q gi|46421796|gb|CN442532.1|CN442532 ssalrgb523282\_rev\_rr0 mixed\_tissue Salmo salar cDNA, mRNA sequence  
R gi|117836218|gb|EG908914.1|EG908914 EST\_ssal\_evf\_11180 ssalevf mixed\_tissue Salmo salar cDNA Salmo salar cDNA clone ssal\_evf\_513\_141\_fwd 3', mRNA sequence  
S gi|117473540|gb|EG805759.1|EG805759 EST\_ssal\_evd\_6503 ssalevd thymus Salmo salar cDNA Salmo salar cDNA clone ssal\_evd\_507\_153\_fwd 3', mRNA sequence  
T gi|117846465|gb|EG919161.1|EG919161 EST\_ssal\_evf\_58143 ssalevf mixed\_tissue Salmo salar cDNA Salmo salar cDNA clone ssal\_evf\_578\_033\_fwd 3', mRNA sequence  
U gi|117452682|gb|EG784901.1|EG784901 EST\_ssal\_evd\_2677 ssalevd thymus Salmo salar cDNA Salmo salar cDNA clone ssal\_evd\_502\_074\_fwd 3', mRNA sequence  
V gi|117445298|gb|EG777521.1|EG777521 EST\_ssal\_evd\_4152 ssalevd thymus Salmo salar cDNA Salmo salar cDNA clone ssal\_evd\_504\_075\_fwd 3', mRNA sequence  
W gi|84971148|gb|DW469549.1|DW469549 SGP297886 Atlantic salmon Testis cDNA library Salmo salar cDNA clone MG4-2678 5', mRNA sequence  
X gi|117832840|gb|EG905536.1|EG905536 EST\_ssal\_evf\_8139 ssalevf mixed\_tissue Salmo salar cDNA Salmo salar cDNA clone ssal\_evf\_509\_088\_fwd 3', mRNA sequence  
Y gi|46421793|gb|CN442529.1|CN442529 ssalkld004026\_rr0 kidney Salmo salar cDNA, mRNA sequence  
Z gi|117862637|gb|EG935333.1|EG935333 EST\_ssal\_evf\_19367 ssalevf mixed\_tissue Salmo salar cDNA Salmo salar cDNA clone ssal\_evf\_524\_164\_fwd 3', mRNA sequence  
a gi|76595600|gb|DV107271.1|DV107271 SGP282765 Atlantic salmon intestine SSH cDNA library Salmo salar cDNA clone ITN-0249, mRNA sequence  
b gi|117863436|gb|EG936132.1|EG936132 EST\_ssal\_evf\_20087 ssalevf mixed\_tissue Salmo salar cDNA Salmo salar cDNA clone ssal\_evf\_525\_155\_fwd 3', mRNA sequence  
c gi|45307330|gb|CK877699.1|CK877699 SGP139032 Atlantic salmon Gills cDNA library Salmo salar cDNA clone G6-1072 5', mRNA sequence  
d gi|117863025|gb|EG935721.1|EG935721 EST\_ssal\_evf\_19716 ssalevf mixed\_tissue Salmo salar cDNA Salmo salar cDNA clone ssal\_evf\_524\_345\_fwd 3', mRNA sequence  
e gi|117833365|gb|EG906061.1|EG906061 EST\_ssal\_evf\_8613 ssalevf mixed\_tissue Salmo salar cDNA Salmo salar cDNA clone ssal\_evf\_509\_331\_fwd 3', mRNA sequence  
f gi|45324069|gb|CK894336.1|CK894336 SGP159941 Atlantic salmon Spleen cDNA library Salmo salar cDNA clone MI5-0365 5', mRNA sequence  
g gi|117468025|gb|EG800244.1|EG800244 EST\_ssal\_evd\_57003 ssalevd thymus Salmo salar cDNA Salmo salar cDNA clone ssal\_evd\_576\_338\_fwd 3', mRNA sequence  
h gi|15844173|gb|BG934345.1|BG934345 SK1-0644 Atlantic Salmon kidney Salmo salar cDNA clone SK1-0644 similar to MHCII beta chain, mRNA sequence  
i gi|117830079|gb|EG902775.1|EG902775 EST\_ssal\_evf\_41399 ssalevf mixed\_tissue Salmo salar cDNA Salmo salar cDNA clone ssal\_evf\_555\_099\_fwd 3', mRNA sequence  
j gi|117479050|gb|EG811267.1|EG811267 EST\_ssal\_evd\_31460 ssalevd thymus Salmo salar cDNA Salmo salar cDNA clone ssal\_evd\_541\_221\_fwd 3', mRNA sequence  
k gi|119020734|gb|EG355035.2|EG355035 Ss\_Gill\_92G11\_M13 Gill SSH library Salmo salar cDNA clone Ss\_Gill\_92G11 5', mRNA sequence  
l gi|24464130|gb|BQ036749.1|BQ036749 SS2-0462 Atlantic Salmon spleen SSH Salmo salar cDNA clone SS2-0462 similar to MHC Class IIB 3'UTR, mRNA sequence  
m gi|117567755|gb|EG888731.1|EG888731 EST\_ssal\_evf\_46758 ssalevf mixed\_tissue Salmo salar cDNA Salmo salar cDNA clone ssal\_evf\_562\_186\_fwd 3', mRNA sequence  
n gi|84981080|gb|DW531430.1|DW531430 EST\_ssal\_plnb\_66 plnb Salmo salar cDNA clone ssal\_plnb\_002\_051\_rev 5', mRNA sequence  
o gi|24464152|gb|BQ036771.1|BQ036771 SS3-0028 Atlantic Salmon spleen SSH Salmo salar cDNA clone SS3-0028 similar to MHC Class IIB 3'UTR, mRNA sequence  
p gi|117855408|gb|EG928104.1|EG928104 EST\_ssal\_evf\_30451 ssalevf mixed\_tissue Salmo salar cDNA Salmo salar cDNA clone ssal\_evf\_540\_161\_fwd 3', mRNA sequence  
q gi|46186995|gb|CN181221.1|CN181221 SGP169437 Atlantic salmon Gills cDNA library Salmo salar cDNA clone GH6-0157 5', mRNA sequence  
r gi|117841433|gb|EG914129.1|EG914129 EST\_ssal\_evf\_15874 ssalevf mixed\_tissue Salmo salar cDNA Salmo salar cDNA clone ssal\_evf\_519\_285\_fwd 3', mRNA sequence  
s gi|117821547|gb|EG894243.1|EG894243 EST\_ssal\_evf\_51720 ssalevf mixed\_tissue Salmo salar cDNA Salmo salar cDNA clone ssal\_evf\_569\_092\_fwd 3', mRNA sequence  
t gi|117441248|gb|EG773471.1|EG773471 EST\_ssal\_evd\_48908 ssalevd thymus Salmo salar cDNA Salmo salar cDNA clone ssal\_evd\_565\_295\_fwd 3', mRNA sequence  
u gi|117833298|gb|EG905994.1|EG905994 EST\_ssal\_evf\_8552 ssalevf mixed\_tissue Salmo salar cDNA Salmo salar cDNA clone ssal\_evf\_509\_300\_fwd 3', mRNA sequence  
v gi|117843378|gb|EG916074.1|EG916074 EST\_ssal\_evf\_55363 ssalevf mixed\_tissue Salmo salar cDNA Salmo salar cDNA clone ssal\_evf\_574\_089\_fwd 3', mRNA sequence  
w gi|117532438|gb|EG863883.1|EG863883 EST\_ssal\_eve\_56787 ssaleve thyroid Salmo salar cDNA Salmo salar cDNA clone ssal\_eve\_577\_148\_fwd 3', mRNA sequence  
x gi|29312862|gb|CB501636.1|CB501636 ssalga504238 head Salmo salar cDNA, mRNA sequence  
y gi|22033569|gb|AJ425401.1|AJ425401 AJ425401 Salmo salar spleen adult Salmo salar cDNA clone s12E01, mRNA sequence  
z gi|22033184|gb|AJ425016.1|AJ425016 AJ425016 Salmo salar spleen adult Salmo salar cDNA clone s02A03, mRNA sequence  
A gi|117460375|gb|EG792594.1|EG792594 EST\_ssal\_evd\_12655 ssalevd thymus Salmo salar cDNA Salmo salar cDNA clone ssal\_evd\_515\_304\_fwd 3', mRNA sequence  
B gi|117837876|gb|EG910572.1|EG910572 EST\_ssal\_evf\_12673 ssalevf mixed\_tissue Salmo salar cDNA Salmo salar cDNA clone ssal\_evf\_515\_164\_fwd 3', mRNA sequence  
C gi|117837875|gb|EG910571.1|EG910571 EST\_ssal\_evf\_12672 ssalevf mixed\_tissue Salmo salar cDNA Salmo salar cDNA clone ssal\_evf\_515\_164\_rev 5', mRNA sequence  
D gi|29313857|gb|CB502631.1|CB502631 ssalmge503052 gut Salmo salar cDNA, mRNA sequence  
E gi|117452671|gb|EG784890.1|EG784890 EST\_ssal\_evd\_2676 ssalevd thymus Salmo salar cDNA Salmo salar cDNA clone ssal\_evd\_502\_074\_rev 5', mRNA sequence  
F gi|117471388|gb|EG803607.1|EG803607 EST\_ssal\_evd\_766 ssalevd thymus Salmo salar cDNA Salmo salar cDNA clone ssal\_evd\_005\_055\_rev 5', mRNA sequence  
G gi|117862328|gb|EG935024.1|EG935024 EST\_ssal\_evf\_19089 ssalevf mixed\_tissue Salmo salar cDNA Salmo salar cDNA clone ssal\_evf\_524\_017\_rev 5', mRNA sequence  
H gi|117538652|gb|EG870097.1|EG870097 EST\_ssal\_eve\_18356 ssaleve thyroid Salmo salar cDNA Salmo salar cDNA clone ssal\_eve\_525\_007\_rev 5', mRNA sequence  
I gi|84981077|gb|DW531427.1|DW531427 EST\_ssal\_plnb\_63 plnb Salmo salar cDNA clone ssal\_plnb\_002\_049\_rev 5', mRNA sequence  
J gi|117832841|gb|EG905537.1|EG905537 EST\_ssal\_evf\_8140 ssalevf mixed\_tissue Salmo salar cDNA Salmo salar cDNA clone ssal\_evf\_509\_088\_rev 5', mRNA sequence  
K gi|117863591|gb|EG936287.1|EG936287 EST\_ssal\_evf\_20226 ssalevf mixed\_tissue Salmo salar cDNA Salmo salar cDNA clone ssal\_evf\_525\_227\_rev 5', mRNA sequence  
L gi|117863592|gb|EG936288.1|EG936288 EST\_ssal\_evf\_20227 ssalevf mixed\_tissue Salmo salar cDNA Salmo salar cDNA clone ssal\_evf\_525\_227\_fwd 3', mRNA sequence  
M gi|29311763|gb|CB500537.1|CB500537 ssalga503184 head Salmo salar cDNA, mRNA sequence  
N gi|117839579|gb|EG912275.1|EG912275 EST\_ssal\_evf\_14205 ssalevf mixed\_tissue Salmo salar cDNA Salmo salar cDNA clone ssal\_evf\_517\_197\_fwd 3', mRNA sequence  
O gi|117839580|gb|EG912276.1|EG912276 EST\_ssal\_evf\_14206 ssalevf mixed\_tissue Salmo salar cDNA Salmo salar cDNA clone ssal\_evf\_517\_197\_rev 5', mRNA sequence  
P gi|117833297|gb|EG905993.1|EG905993 EST\_ssal\_evf\_8551 ssalevf mixed\_tissue Salmo salar cDNA Salmo salar cDNA clone ssal\_evf\_509\_300\_rev 5', mRNA sequence  
Q gi|56732318|gb|CX153912.1|CX153912 ssalsla009080\_0 spleen Salmo salar cDNA, mRNA sequence  
R gi|29314089|gb|CB502863.1|CB502863 ssalmgd501014 gut Salmo salar cDNA, mRNA sequence  
S gi|29312949|gb|CB501723.1|CB501723 ssalga508186 head Salmo salar cDNA, mRNA sequence  
T gi|117532439|gb|EG863884.1|EG863884 EST\_ssal\_eve\_56788 ssaleve thyroid Salmo salar cDNA Salmo salar cDNA clone ssal\_eve\_577\_148\_rev 5', mRNA sequence  
U gi|29325286|gb|CB514060.1|CB514060 ssalrgb548118 mixed\_tissue Salmo salar cDNA, mRNA sequence  
V gi|29313534|gb|CB502308.1|CB502308 ssalmge501121 gut Salmo salar cDNA, mRNA sequence  
W gi|117860323|gb|EG933019.1|EG933019 EST\_ssal\_evf\_18531 ssalevf mixed\_tissue Salmo salar cDNA Salmo salar cDNA clone ssal\_evf\_523\_116\_fwd 3', mRNA sequence  
X gi|117860334|gb|EG933030.1|EG933030 EST\_ssal\_evf\_18532 ssalevf mixed\_tissue Salmo salar cDNA Salmo salar cDNA clone ssal\_evf\_523\_116\_rev 5', mRNA sequence  
Y gi|117473539|gb|EG805758.1|EG805758 EST\_ssal\_evd\_6502 ssalevd thymus Salmo salar cDNA Salmo salar cDNA clone ssal\_evd\_507\_153\_rev 5', mRNA sequence  
Z gi|24352046|gb|CA045876.1|CA045876 ssalpitl501132 head Salmo salar cDNA, mRNA sequence  
a gi|117846464|gb|EG919160.1|EG919160 EST\_ssal\_evf\_58142 ssalevf mixed\_tissue Salmo salar cDNA Salmo salar cDNA clone ssal\_evf\_578\_033\_rev 5', mRNA sequence  
b gi|84981078|gb|DW531428.1|DW531428 EST\_ssal\_plnb\_64 plnb Salmo salar cDNA clone ssal\_plnb\_002\_049\_fwd 3', mRNA sequence  
c gi|29317226|gb|CB506000.1|CB506000 ssalmge502087 gut Salmo salar cDNA, mRNA sequence  
d gi|29312719|gb|CB501493.1|CB501493 ssalga507383 head Salmo salar cDNA, mRNA sequence  
e gi|25998000|gb|CA768745.1|CA768745 ssalga002009 head Salmo salar cDNA, mRNA sequence  
f gi|84984038|gb|DW534388.1|DW534388 EST\_ssal\_plnb\_3024 plnb Salmo salar cDNA clone ssal\_plnb\_020\_186\_fwd 3', mRNA sequence  
g gi|56732584|gb|CX154049.1|CX154049 ssalsla011018\_0 spleen Salmo salar cDNA, mRNA sequence  
h gi|29311208|gb|CB499982.1|CB499982 ssalga502119 head Salmo salar cDNA, mRNA sequence  
i gi|117843379|gb|EG916075.1|EG916075 EST\_ssal\_evf\_55364 ssalevf mixed\_tissue Salmo salar cDNA Salmo salar cDNA clone ssal\_evf\_574\_089\_rev 5', mRNA sequence  
j gi|29311754|gb|CB500528.1|CB500528 ssalga503157 head Salmo salar cDNA, mRNA sequence  
k gi|56732942|gb|CX154230.1|CX154230 ssalsla011020\_0 spleen Salmo salar cDNA, mRNA sequence  
l gi|29312145|gb|CB500919.1|CB500919 ssalga501087 head Salmo salar cDNA, mRNA sequence  
m gi|117567754|gb|EG888730.1|EG888730 EST\_ssal\_evf\_46757 ssalevf mixed\_tissue Salmo salar cDNA Salmo salar cDNA clone ssal\_evf\_562\_186\_rev 5', mRNA sequence  
n gi|117862638|gb|EG935334.1|EG935334 EST\_ssal\_evf\_19368 ssalevf mixed\_tissue Salmo salar cDNA Salmo salar cDNA clone ssal\_evf\_524\_164\_rev 5', mRNA sequence  
o gi|29311287|gb|CB500061.1|CB500061 ssalga502268 head Salmo salar cDNA, mRNA sequence  
p gi|117538641|gb|EG870086.1|EG870086 EST\_ssal\_eve\_18355 ssaleve thyroid Salmo salar cDNA Salmo salar cDNA clone ssal\_eve\_525\_007\_fwd 3', mRNA sequence  
q gi|117529415|gb|EG861056.1|EG861056 EST\_ssal\_eve\_54241 ssaleve thyroid Salmo salar cDNA Salmo salar cDNA clone ssal\_eve\_573\_343\_rev 5', mRNA sequence  
r gi|117529416|gb|EG861057.1|EG861057 EST\_ssal\_eve\_54242 ssaleve thyroid Salmo salar cDNA Salmo salar cDNA clone ssal\_eve\_573\_343\_fwd 3', mRNA sequence  
s gi|117830078|gb|EG902774.1|EG902774 EST\_ssal\_evf\_41398 ssalevf mixed\_tissue Salmo salar cDNA Salmo salar cDNA clone ssal\_evf\_555\_099\_rev 5', mRNA sequence  
t gi|117855409|gb|EG928105.1|EG928105 EST\_ssal\_evf\_30452 ssalevf mixed\_tissue Salmo salar cDNA Salmo salar cDNA clone ssal\_evf\_540\_161\_rev 5', mRNA sequence  
u gi|29311341|gb|CB500115.1|CB500115 ssalga502376 head Salmo salar cDNA, mRNA sequence  
v gi|117847454|gb|EG920150.1|EG920150 EST\_ssal\_evf\_59033 ssalevf mixed\_tissue Salmo salar cDNA Salmo salar cDNA clone ssal\_evf\_579\_126\_fwd 3', mRNA sequence  
w gi|85028668|gb|DW557324.1|DW557324 EST\_ssal\_rgb2\_21743 rgb2 Salmo salar cDNA clone ssal\_rgb2\_535\_139\_fwd 3', mRNA sequence  
x gi|29313804|gb|CB502578.1|CB502578 ssalmge501344 gut Salmo salar cDNA, mRNA sequence  
y gi|24390927|gb|CA060684.1|CA060684 ssalrgb537125 mixed\_tissue Salmo salar cDNA, mRNA sequence  
z gi|117460374|gb|EG792593.1|EG792593 EST\_ssal\_evd\_12654 ssalevd thymus Salmo salar cDNA Salmo salar cDNA clone ssal\_evd\_515\_304\_rev 5', mRNA sequence  
A gi|117821546|gb|EG894242.1|EG894242 EST\_ssal\_evf\_51719 ssalevf mixed\_tissue Salmo salar cDNA Salmo salar cDNA clone ssal\_evf\_569\_092\_rev 5', mRNA sequence  
B gi|117863437|gb|EG936133.1|EG936133 EST\_ssal\_evf\_20088 ssalevf mixed\_tissue Salmo salar cDNA Salmo salar cDNA clone ssal\_evf\_525\_155\_rev 5', mRNA sequence  
C gi|117841435|gb|EG914131.1|EG914131 EST\_ssal\_evf\_15875 ssalevf mixed\_tissue Salmo salar cDNA Salmo salar cDNA clone ssal\_evf\_519\_285\_rev 5', mRNA sequence  
D gi|117831016|gb|EG903712.1|EG903712 EST\_ssal\_evf\_42243 ssalevf mixed\_tissue Salmo salar cDNA Salmo salar cDNA clone ssal\_evf\_556\_160\_fwd 3', mRNA sequence  
E gi|117831017|gb|EG903713.1|EG903713 EST\_ssal\_evf\_42244 ssalevf mixed\_tissue Salmo salar cDNA Salmo salar cDNA clone ssal\_evf\_556\_160\_rev 5', mRNA sequence  
F gi|24353545|gb|CA047375.1|CA047375 ssalga002058 head Salmo salar cDNA, mRNA sequence  
G gi|24343605|gb|CA042685.1|CA042685 ssalmge002035 gut Salmo salar cDNA, mRNA sequence  
H gi|24354569|gb|CA048399.1|CA048399 ssalklc001042 kidney Salmo salar cDNA, mRNA sequence  
I gi|25997675|gb|CA768420.1|CA768420 ssalga007046 head Salmo salar cDNA, mRNA sequence  
J gi|25996980|gb|CA767725.1|CA767725 ssalplnb504207 gut Salmo salar cDNA, mRNA sequence  
K gi|24344625|gb|CA043705.1|CA043705 ssalplnb510108 gut Salmo salar cDNA, mRNA sequence  
L gi|29311834|gb|CB500608.1|CB500608 ssalga503253 head Salmo salar cDNA, mRNA sequence  
M gi|117833366|gb|EG906062.1|EG906062 EST\_ssal\_evf\_8614 ssalevf mixed\_tissue Salmo salar cDNA Salmo salar cDNA clone ssal\_evf\_509\_331\_rev 5', mRNA sequence  
N gi|117479048|gb|EG811265.1|EG811265 EST\_ssal\_evd\_31459 ssalevd thymus Salmo salar cDNA Salmo salar cDNA clone ssal\_evd\_541\_221\_rev 5', mRNA sequence  
O gi|117863026|gb|EG935722.1|EG935722 EST\_ssal\_evf\_19717 ssalevf mixed\_tissue Salmo salar cDNA Salmo salar cDNA clone ssal\_evf\_524\_345\_rev 5', mRNA sequence  
P gi|29312880|gb|CB501654.1|CB501654 ssalga504294 head Salmo salar cDNA, mRNA sequence  
Q gi|29316781|gb|CB505555.1|CB505555 ssalmgd503197 gut Salmo salar cDNA, mRNA sequence  
R gi|117441249|gb|EG773472.1|EG773472 EST\_ssal\_evd\_48909 ssalevd thymus Salmo salar cDNA Salmo salar cDNA clone ssal\_evd\_565\_295\_rev 5', mRNA sequence  
S gi|29319125|gb|CB507479.1|CB507479 ssaltc023073\_rev reproductive Salmo salar cDNA, mRNA sequence  
T gi|24355294|gb|CA049124.1|CA049124 ssalkspa004066 kidney Salmo salar cDNA, mRNA sequence  
U gi|25998300|gb|CA769045.1|CA769045 ssalksha001065 kidney Salmo salar cDNA, mRNA sequence  
V gi|45650058|gb|CK991089.1|CK991089 ssalrgb523347 mixed\_tissue Salmo salar cDNA, mRNA sequence  
W gi|56732452|gb|CX153980.1|CX153980 ssalsla004092\_0 spleen Salmo salar cDNA, mRNA sequence  
X gi|117472192|gb|EG804411.1|EG804411 EST\_ssal\_evd\_53032 ssalevd thymus Salmo salar cDNA Salmo salar cDNA clone ssal\_evd\_571\_182\_fwd 3', mRNA sequence  
Y gi|117472203|gb|EG804422.1|EG804422 EST\_ssal\_evd\_53033 ssalevd thymus Salmo salar cDNA Salmo salar cDNA clone ssal\_evd\_571\_182\_rev 5', mRNA sequence  
Z gi|24392032|gb|CA061789.1|CA061789 ssalrgp001040 mixed\_tissue Salmo salar cDNA, mRNA sequence  
a gi|29315318|gb|CB504092.1|CB504092 ssalema003060 gut Salmo salar cDNA, mRNA sequence  
b gi|25999296|gb|CA770041.1|CA770041 ssalrgb509310 mixed\_tissue Salmo salar cDNA, mRNA sequence  
c gi|25999805|gb|CA770550.1|CA770550 ssalob008043 reproductive Salmo salar cDNA, mRNA sequence  
d gi|59636903|gb|DN047911.1|DN047911 ssallksd014085\_0 mixed\_tissue\_SDb Salmo salar cDNA, mRNA sequence  
e gi|57011057|gb|CX281969.1|CX281969 ssalkld003028\_0 kidney Salmo salar cDNA, mRNA sequence  
f gi|57010809|gb|CX282299.1|CX282299 ssalkld010073\_0 kidney Salmo salar cDNA, mRNA sequence  
g gi|29316790|gb|CB505564.1|CB505564 ssalmge504259 gut Salmo salar cDNA, mRNA sequence  
h gi|24381598|gb|CA051355.1|CA051355 ssalrga501053 mixed\_tissue Salmo salar cDNA, mRNA sequence  
i gi|24343965|gb|CA043045.1|CA043045 ssalmgf005024 gut Salmo salar cDNA, mRNA sequence

9 SNPs detected

A B C D E F G H I J K L M N O P Q R S T U V W X Y Z a b c d e f g h i j k l m n o p q r s t u v w x y z A B C D E F G H I J K L M N O P Q R S T U V W X Y Z a b c d e f g h i j k l m n o p q r s t u v w x y z A B C D E F G H I J K L M N O P Q R S T U V W X Y Z a b c d e f g h i  cosegregation weighted

482 . . . . . . . . T T T C C C T T T T T T T T C T T T . T . T T C T C T . . . T . . T . T . . . . . . . . . . . . . . . . . . . . . . . . . . . . . . . . . . . . . . . . . . . . . . . . . . . . . . . . . . . . . . . . . . . . . . . . . . . . . . . . . . . . . . . . . . . . . . .   7/9 15.67
948 . . . . . . . . . . . . . . . . . . . . . . . . . . . . . . . T T T T T T T T T T T T T T T T T T T C T T T T T T . T T T T T T T T T T T T T T T T T T T . . T T T T T T T T T T T T T T T T T T T T C C C C T T T T T T T T C T T T T T T T T T T . . . . . . . T T T T T C C T T T   1/9 7.83
1009 . . . . . . . . . . . . . . . . . . . . . . . . . . . . . . . . . . - - T T - - T - - - - T - - - - - - - - - - T . - - - - - - - - - - - - - - T T - - - - - - - - - - - - - - - - - - - - - - - - - - - - - - - - - - - - - - - - - - - - - - T T . . . . . . . T T T T T T T T T T   7/9 54.28
1010 . . . . . . . . . . . . . . . . . . . . . . . . . . . . . . . . . . - - A A - - A - - - - A - - - - - - - - - - A . - - - - - - - - - - - - - - A A - - - - - - - - - - - - - - - - - - - - - - - - - - - - - - - - - - - - - - - - - - - - - - A A . . . . . . . A A A A A A A A A A   7/9 54.28
1011 . . . . . . . . . . . . . . . . . . . . . . . . . . . . . . . . . . - - C C - - C - - - - C - - - - - - - - - - C . - - - - - - - - - - - - - - C C - - - - - - - - - - - - - - - - - - - - - - - - - - - - - - - - - - - - - - - - - - - - - - C C . . . . . . . C C C C C C C C C C   7/9 54.28
1012 . . . . . . . . . . . . . . . . . . . . . . . . . . . . . . . . . . - - C C - - C - - - - C - - - - - - - - - - C . - - - - - - - - - - - - - - C C - - - - - - - - - - - - - - - - - - - - - - - - - - - - - - - - - - - - - - - - - - - - - - C C . . . . . . . C C C C C C C C C C   7/9 54.28
1013 . . . . . . . . . . . . . . . . . . . . . . . . . . . . . . . . . . - - T T - - T - - - - T - - - - - - - - - - T . - - - - - - - - - - - - - - T T - - - - - - - - - - - - - - - - - - - - - - - - - - - - - - - - - - - - - - - - - - - - - - T T . . . . . . . T T T T T T T T T T   7/9 54.28
1077 . . . . . . . . . . . . . . . . . . . . . . . . . . . . . . . . . . G . . T T T A T A T T A T T T T A T T T T T A . T T T T T T T T T T T T T T A A T T T T T T T T T T T T T T T T T T T T T T T T T T A A A T T T T T T T T T T T T T T T T T A A . . . . . A A A A A A T T T A A A   1/9 7.75
1318 . . . . . . . . . . . . . . . . . . . . . . . . . . . . . . . . . . . . . . . . . . . . . . . . . . . A A G G A A A A A A A A A A A A A A A A A A A A A A A A A A A A A A A A A A A A A A A A A A A A A A A A A A A A A A A A G A A A A A A A A A A . A A A A A A A A A A G G G A A A   7/9 48.68
